# Attitudes of non-participating general practitioners and community pharmacists towards interprofessional medication management in primary care: an interview study

**DOI:** 10.1007/s11096-022-01434-3

**Published:** 2022-10-08

**Authors:** Robert Moecker, Andreas Fuchs, ARMIN Study Group, Christiane Eickhoff, Uta Mueller, Martin Schulz, Andreas Fuchs, Dorit Braun, Ulf Maywald, Catharina Doehler, Mike Maetzler, Anja Auerbach, Urs Dieter Kuhn, Anke Moeckel, Christine Honscha, Susanne Donner, Stefan Fink, Kathrin Wagner, Andreas D. Meid, Robert Moecker, Carmen Ruff, Hanna M. Seidling, Felicitas Stoll, Marina Weissenborn, Lucas Wirbka, Walter E. Haefeli, Marina Weissenborn, Hanna M. Seidling

**Affiliations:** 1 Department of Clinical Pharmacology and Pharmacoepidemiology, Heidelberg University Hospital, Im Neuenheimer Feld 410, 69120 Heidelberg, Germany; 2 Cooperation Unit Clinical Pharmacy, University of Heidelberg, Im Neuenheimer Feld 410, 69120 Heidelberg, Germany; 3 AOK PLUS – Die Gesundheitskasse, Sternplatz 7, 01067 Dresden, Germany

**Keywords:** Interprofessional collaboration, Medication management, Medication review, Medication safety, Primary care

## Abstract

**Background:**

Interprofessional medication management in primary care is a recognized strategy for improving medication safety, but it is poorly implemented in Germany. As a pilot project, ARMIN [Arzneimittelinitiative Sachsen-Thüringen] was initiated in 2014 to establish better interprofessional medication management between general practitioners and community pharmacists.

**Aim:**

The aim of this study was to explore the views of non-participating general practitioners and community pharmacists towards interprofessional medication management within ARMIN and to identify barriers to participation.

**Method:**

This was an interview study comprising a series of semi-structured telephone interviews. In total, 36 general practitioners and 15 community pharmacists were interviewed in the period between March and June 2020. Data were analyzed using thematic analysis as an inductive approach and the consolidated framework for implementation research as a deductive approach.

**Results:**

Many general practitioners and community pharmacists had a generally positive attitude towards interprofessional medication management. However, various barriers were identified and categorized into five major themes: (I) collaboration between general practitioners and community pharmacists, e.g. concerning general practitioners’ professional sovereignty and pharmacists’ fear of jeopardizing their relationship with general practitioners when interfering in therapy; (II) eligibility for participation, e.g., the fact that patients had to be insured with a specific statutory health insurance fund; (III) local circumstances, e.g. many pharmacists could not find a collaborating general practitioner (and vice versa). Moreover, patient demand was low, probably because patients were not aware of the program; (IV) information technology, e.g. concerning the lack of available software and data security concerns; and (V) cost–benefit ratio, e.g. the fact that potential benefits were outweighed by program-associated costs.

**Conclusion:**

The perceived discrepancy between positive attitudes and multiple prevalent barriers indicates considerable potential for further interprofessional collaboration between general practitioners and community pharmacists.

**Supplementary Information:**

The online version contains supplementary material available at 10.1007/s11096-022-01434-3.

## Impact Statements

- Finding a collaborating partner in a medication management program needs facilitation, as it was a key barrier for many pharmacists and some general practitioners.
- Besides general practitioners and community pharmacists, patients also need to be informed about new such programs. Potential benefits should be highlighted and incentives used to foster patient demand.
- The perceived gap between healthcare professionals' positive attitudes and reported barriers suggests considerable room for improvement.

## Introduction

According to the Joint Commission of Pharmacy Practitioners in the United States, Medication Management comprises a broad range of professional services, which are “patient-centered, pharmacist-provided, collaborative services that focus on medication appropriateness, effectiveness, safety, and adherence with the goal of improving health outcomes” [1, 2]. These services can improve medication appropriateness, adherence, clinical outcomes, and reduce the number of hospital (re)admissions in discharged or multimorbid patients with polypharmacy [3–6]. Although already established in countries such as Australia and the Unites States [7–9], they are not yet comprehensively established in many European countries [10].

In Germany, medication management is not yet available at the national level. However, several programs such as the ARMIN [Arzneimittelinitiative Sachsen-Thüringen] Medication Management Program (MMP) were launched at the regional level, with the aim of increasing medication safety among home-dwelling patients with polypharmacy, e.g., by reducing drug-related problems (DRPs) as well as increasing adherence [11].

In Germany, pharmacies are owner-operated (up to four per owner) and their local computer systems are generally poorly connected with those of other healthcare professionals (HCPs) (including those of other pharmacists) [12]. Although there is a national digital health strategy, no regular electronic health records and no electronic health information exchange was in place at the time of the ARMIN project. There were also no national initiatives established in which community pharmacists (CPs) and other HCPs in primary care could collaborate on a regular basis, such as in the UK or in the US [13, 14]. However, many physicians offer disease management programs. Further, national guidelines increasingly recommend involving pharmacists in the therapy of patients [15, 16]. Around 88% of the patients in Germany are insured with a statutory health insurance (SHI) fund which pays for healthcare utilization.

### The ARMIN project

ARMIN is an interprofessional, electronically supported project that was launched in the German federal states of Saxony and Thuringia. It was developed, implemented, and remunerated by the SHI fund *AOK PLUS,* the Federal Union of German Associations of Pharmacists, the Association of Statutory Health Insurance Physicians—Saxony, and the Association of Statutory Health Insurance Physicians—Thuringia. ARMIN consists of three components that were implemented consecutively: preferred generic prescribing (instead of brand name products), preferred prescribing of first-line drugs according to a medication formulary (both since 2014), and MMP (since 2016). The MMP consists of an initial interprofessional medication review and up to three follow-up appointments per year. Patients had to enroll in the MMP in order to receive this service. CPs and general practitioners (GPs) had to register for the MMP in order to deliver this service. Although GPs and CPs could register individually, both the patient’s GP and the CP needed to participate in the MMP in order to enroll patients and to provide the service. Once HCPs had registered, they needed to install required hardware and software to connect their local computer systems to a central medication data server which allowed GPs and CPs to both exchange patent data and communicate digitally in the MMP.

The MMP defines specific tasks for CPs and GPs in accordance with their pharmaceutical and medical expertise, respectively. First, the CP conducted a brown bag review and reconciled the medication data from different sources, e.g., patient interview, medication list, and claims data. Then the CP discussed any discrepancies as well as any DRPs, such as side effects, with the patient. Following this, the CP forwarded the results of the pharmaceutical assessment, e.g. side effects, drug-drug interaction, and duplicate medications, together with the preliminary medication list to the GP. The GP conducted the medical assessment, such as checking diagnoses and clinical parameters and adjusting dosages. Next, the GP uploaded the completed medication list to the medication data server and both GP and CP agreed on any actions regarding changes of the patient’s therapy. After the initial medication review, both HCPs monitored the patient’s medication and conducted further assessments whenever the patient’s medication changed. All changes in medication, corresponding DRPs, and any further remarks were communicated between the GP and the CP via the medication data server [12, 17]. GPs and CPs received approximately 100 EUR and 25 EUR for the initial medication review and follow-up interview, respectively.

Although the implementation of medication reviews has increased in recent years [10], in ARMIN, only about 15.8% (243/1536) of the CPs and 3.9% (165/4178) of the GPs in Saxony and Thuringia had registered for the MMP before the beginning of 2020. As MMP are complex services [18], their implementation still faces several barriers such as attitudes towards the service, lack of time, lack of (trained) staff, or increased workload [19–22]. However, these barriers are often derived from studies in which HCPs were already trying to implement MMP. HCPs who are slower to adopt new services experience barriers at the very beginning when implementing a service [23], which might even extend to perceived barriers pre-implementation. Furthermore, HCP who adopt new services comparably quickly might have more positive attitudes and report fewer barriers because they can solve barriers autonomously, as studies have shown [24]. Hence, our study investigates the attitudes of GPs and CPs and their experiences of barriers prior to participating in the interprofessional MMP.

### Aim

The aim of this study was to explore the views of non-participating general practitioners and community pharmacists towards interprofessional medication management within ARMIN and to identify barriers to participation.

### Ethics approval

Ethical approval was obtained from the responsible Ethics Committee of the Medical Faculty of Heidelberg University (reference no.: S-142/2019) on January 9, 2020. All interviewees participated voluntarily and gave their informed consent before being included in this study.

## Method

Semi-structured telephone interviews were conducted with GPs and CPs to explore their attitudes towards the MMP and to identify barriers that have kept them from participating. Wherever applicable, the study is reported according to the consolidated criteria for reporting qualitative research (COREQ) [25].

### Participants and recruitment

GPs and CPs were eligible if they were not participating in the medication management but had already used preferred generic prescribing. Familiarity with preferred generic prescribing was chosen as an inclusion criterion because the views of these HCPs were expected to be more informative than of HCPs, who had no touchpoints with ARMIN at all. It was expected that HCPs with no touchpoints with ARMIN, meaning that they had not used preferred generic prescribing, would have little or even no knowledge about the ARMIN project.

The initial goal was to interview 35 GPs and 15 CPs. Recruitment took place between February and May 2020. A higher recruitment goal for GPs was chosen because fewer GPs than CPs participated in ARMIN and it was anticipated that GPs encountered more barriers during the implementation of ARMIN.

First, potential participants, i.e. GPs and CPs who were not participating in the medication management and who had already used preferred generic prescribing, were identified by AOK PLUS (responsible SHI fund). In total, the SHI fund identified 240 GPs and 200 CPs as potential participants. Of these, 35 GPs and 15 CPs were initially recruited by the SHI fund via telephone using random sampling. After giving their consent, the participants’ contact information was sent to the research team at the University of Heidelberg. The research team consisted of RM, MW, LM, and HMS.

Second, participants were contacted by RM. RM referred to the initial contact of the SHI fund, explained the purpose of the study in more detail and the funding source (i.e. SHI fund) and introduced the research team to the participants while also emphasizing the independence and unbiased judgement of the researchers. Furthermore, the procedure of the semi-structured telephone interview was explained and an estimate for the duration of the interview was provided. If HCPs were still willing to participate, an appointment was scheduled for the interview.

Third, not all HCPs who agreed to participate when contacted by the SHI fund were still willing to participate in this study when contacted by the researchers. In total, the SHI fund had to approach additional 28 GPs and 6 additional CPs and sent their contact information to the research team to reach the recruitment goal. Reasons for refusing to participate in this study when contacted by the researchers were mostly time-related because of the arrival of the COVID-19 pandemic.

### Interview guide

A pool of interview questions was built on the basis of a previous literature search and the domains of CFIR [26]. The CFIR is a well-established framework for investigating barriers and facilitators when implementing research projects and comprises the following five domains: “intervention characteristics”, “outer setting”, “inner setting”, “characteristics of individuals”, and “process”. Interview questions were developed with regard to the different domains of CFIR and adapted to the ARMIN MMP. On the basis of their applicability to the ARMIN project in general and the MMP in particular, interview questions were selected from a pool of interview questions. Fellow experts who were involved in planning and implementing the ARMIN project were contacted for their feedback and the interview guide was revised in an iterative process. Question comprehensibility and interview structure and length were pilot tested with one GP and two CPs.

The interview guide (see supplementary material 1) contained three sections. To contextualize their subsequent responses, participants were first asked to explain what they knew in general about the ARMIN project. Second, they were asked what they regarded as positive and negative aspects of the ARMIN project. Third, distinct barriers regarding the different components in ARMIN and beliefs about the impact of the MMP on patient care, workflows, and collaboration between GPs and CPs were presented as statements to the participants (see Table 2). Participants were asked to respond using a 3-Likert scale, with the options being “agree”, “undecided”, and “disagree”, and to explain their answers in more detail.

### Data collection

The interviews were conducted between March and June 2020 and by the same researcher (RM, male, pharmacist, doctoral student) in order to minimize the risk of interviewer bias. Interviews were initially supervised by MW (female, pharmacist, senior researcher) and HMS (female, pharmacist, doctoral degree, group leader), both of whom had considerable experience in qualitative research, and after each interview a debriefing took place. Interviews were not recorded because it would have been a further hurdle for HCPs to participate while at the same time making a protocol of specific prompts and HCPs’ responses. Instead, all interviews were protocolled by a second researcher, i.e., MW, HMS, or LM (female, post-graduate student). Data were collected using a piloted template which consisted of checkboxes for Likert scale questions and blank boxes for open-ended questions and additional prompts. Towards the end of each interview, the researchers quickly summarized the findings and presented them to the interviewee. Interviewees were asked whether they had any remarks or would like to add anything, e.g., barriers which were not addressed in the interview. Most participants were at work when they were interviewed.

Immediately after each interview, the researchers compared, discussed, and consolidated their notes. All interviewees gave their permission to be contacted again if further questions arose during data analysis. However, it was not necessary to contact the respondents again.

### Data analysis

Absolute and relative frequencies of the number of respondents who agreed with the presented statements were quantified. Then, consolidated protocols were re-read and attitudes and barriers were labeled with “codes”. As new codes emerged during the interviews, they were continuously refined and checked against the data from the interview protocols. Data saturation was defined as no new codes emerged in the three consecutive interviews with GPs or CPs and reached according to the recruitment goal. Similar codes were categorized by theme using an inductive approach which employed thematic analysis [27, 28]. Categorized codes and generated themes were checked by a second researcher. Differences were discussed and the results refined. Finally, in a deductive approach the emerged codes were categorized using the five domains and corresponding constructs of CFIR [26, 29] to complement the thematic analysis and increase comparability with other studies [30–32]. Microsoft Office 2016 was used to facilitate data analysis.

## Results

In total, 36 GPs and 15 CPs took part in the study. The interviews lasted 33 min on average (SD ± 9 min) and took place via telephone. The demographic data of the participants are shown in Table 1.

**Table 1:** Characteristics of the participants

	General practitioners (N = 36)	Community pharmacists (N = 15)
Gender		
Male	15 (41.7%)	8 (53.3%)
Female	21 (58.3%)	7 (46.7%)
Region		
Saxony	17 (47.2%)	9 (60.0%)
Thuringia	19 (52.8%)	6 (40.0%)
Age [years] (mean ± SD; range)	55 (± 9; 36–78)	46 (± 10; 32–66)
Professional experience [years] (mean ± SD; range)	17 (± 10; 1–45)	20 (± 10; 5–38)
Working hours		
Full-time	33 (91.7%)	12 (80.0%)
Part-time	3 (8.3%)	3 (20.0%)
Workplace	Single practice: 29 (80.6%) Joint practice: 3 (8.3%) Medical service center: 4 (11.1%)	Manager: 12 (80.0%) Pharmacy branch manager: 1 (6.7%) Employee: 2 (13.3%)
Previously or currently enrolled in other programs to improve patient care	28 (77.8%)	6 (40.0%)

### Agreement with statements

When GPs and CPs were asked what aspects they valued about ARMIN, most of them highlighted the increase of medication safety and the improvement of interprofessional communication and collaboration. In addition, most interviewees agreed with the presented statements about the ARMIN project (Table 2). However, although most interviewees had rather positive attitudes, various barriers were expressed.

**Table 2:** General agreement of GPs and CPs with statements about ARMIN

No	Statement	Number of respondents agreeing (percentage)	
		General practitioner (N = 36)	Community pharmacist (N = 15)
1	Preferred generic prescription is useful	26 (72.2%)	13 (86.7%)
2	Preferred prescribing of first-line drugs according to a medication formulary is useful	21 (58.3%)	10 (66.7%)
3	Joint medication management (between GPs and CPs) is useful	25 (69.4%)	15 (100.0%)
4	A clear allocation of tasks in medication management between GPs and CPs is useful	33 (91.7%)	15 (100.0%)
5	Electronic communication in medication management between GPs and CPs is useful	24 (66.7%)	15 (100.0%)
6	ARMIN can improve patient care	26 (72.2%)	15 (100.0%)
7	ARMIN is relevant to my daily work routine	32 (88.9%)	14 (93.3%)
8	I know the ARMIN workflows	12 (33.3%)	10 (66.7%)
9	I manage patients who would benefit from medication management	31 (86.1%)	15 (100.0%)
10	I was asked about ARMIN by my patients	0 (0.0%)	1 (6.7%)
11	I was contacted by CPs/GPs because they would like to collaborate within the ARMIN medication management	9 (25.0%)	3 (20.0%)
12	I contacted CPs/GPs because I would like to collaborate within the ARMIN medication management	7 (19.4%)	7 (46.7%)
13	I would like to work with pharmacists/GPs to optimize my patients’ drug therapy	33 (91.7%)	14 (93.3%)
14	I could easily integrate medication management into my daily work routine	16 (44.4%)	6 (40.0%)
15	The remuneration for performing medication management in ARMIN is appropriate	31 (86.1%)	9 (60.0%)
16	The technical implementation of ARMIN seems to be feasible. *	9 (75.0%)	7 (100.0%)
17	I feel that signing separate contracts for programs from different health insurance companies is a limitation	28 (77.8%)	8 (53.3%)

ARMIN: Arzneimittelinitiative Sachsen-Thüringen (medication management program); CP: community pharmacist; GP: general practitioner; HCP: healthcare professional^*^Statement was only rated by HCPs who have already dealt with technical implementation (N = 12 GPs and N = 7 CPs)

### Inductive thematic analysis

**Fig. 1:**
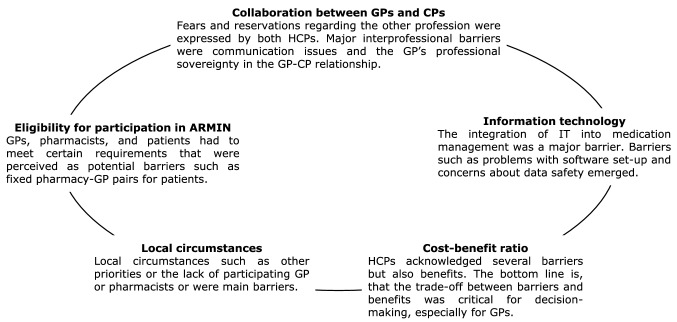
Themes in the participation of ARMIN. *ARMIN*: Arzneimittelinitiative Sachsen-Thüringen (medication management program); *CP*: community pharmacist; *GP*: general practitioner; *HCP*: healthcare professional; *IT*: information technology

When categorizing reported barriers, five themes emerged from the thematic analysis (Fig. 1).

#### Collaboration between GPs and CPs

A key issue was the GPs’ professional sovereignty. On the one hand, GPs reported that they felt monitored by CPs and that CPs interfered in therapy. On the other hand, some CPs feared that they would jeopardize their relationship with the GP if they became more involved in therapy. In addition, poor communication made cooperation even more difficult. CPs often had difficulty in reaching GPs.

#### Eligibility for participation

Some HCPs felt it was a disadvantage that patients needed a fixed GP-CP pair to participate in the ARMIN MMP. In addition, CPs had to be members of the corresponding State Association of Pharmacists. As patients had to be insured with SHI fund AOK PLUS in order to participate, GPs expressed a desire for more standardization among different SHI funds.

#### Local circumstances

The most common barrier for CPs was the lack of GP participation. GPs were neither interested nor did they have the appropriate software to participate in the MMP. Both HCPs reported that the ARMIN MMP was simply another program in patient care for GPs, which is why their motivation to participate was sometimes low.

#### Information technology (IT)

It was particularly difficult for GPs to meet the technical requirements for the ARMIN MMP. In some cases, their software providers did not offer the relevant software modules. In addition, GPs had concerns about data security and the potential susceptibility of their own computer systems to malfunctions.

#### Cost–benefit ratio

Both HCPs frequently perceived the additional time required and the bureaucracy as high. Likewise, additional costs, especially for software and personnel, were perceived as barriers. While the potential benefits of the ARMIN MMP were partially acknowledged, these benefits did not seem to be an added value for HCPs which already offered MMP.

### Deductive analysis (CFIR)

Table 3 summarizes the identified barriers according to CFIR and provides exemplary excerpts of the notes taken during the semi-structured interviews. Among GPs, frequently reported barriers in descending order of frequency were expenditure of time, problems with software set-up, other priorities (e.g., take-over of a family practice), concerns about data security, and GPs’ professional sovereignty. Among CPs, frequently reported barriers were the lack of participating GPs, expenditure of time, costs, difficulties in communication and the professional sovereignty of GPs.

**Table 3:** Identified barriers and exemplary excerpts from interviews using CFIR

CFIR domain CFIR construct	Barrier	Explanation	Interview excerpt
I. Intervention characteristics			
Intervention source	n.a		
Evidence strength & quality	Lack of evidence	One GP said, he was not sure if ARMIN could contribute to better patient care because there was no evidence yet	“… not sure if ARMIN is able to improve patient care… further evidence is needed …” (GP-20)
Relative advantage	GPs’ preference for other projects	ARMIN was seen as an additional, separate program and not as a potential extension to already existing programs such as disease management programs	“… electronic prescription and an electronic medication list are about to be implemented nationwide …” (CP-6)
		Other GP programs seem to be better remunerated than the ARMIN MM	
		One CP would rather wait until features such as electronic medication lists will be made available within the currently ongoing digitalization in the German healthcare system (e.g., as part of the digital health act (Deutscher Bundestag, 2019))	
Adaptability	n.a		
Trialability	n.a		
Complexity	Duration of a medication review	For many GPs and few CPs, the allocated time for the whole process of the initial medication review (approx. 90 min) was too long to be feasible in their daily routine. Furthermore, the time to recruit and enroll patients was seen critically by GPs	“… the time that is needed to recruit patients and explain ARMIN… and then some patients don’t even want to participate …” (GP-9)
	Bureaucracy	Some interviewees mentioned a very high bureaucratic burden	“… madness of bureaucracy …” (GP-6)
Design quality and packaging	Troublesome software set-up	GPs as well as CPs had difficulties with setting up the ARMIN-software in their local computer system, e.g., because the software was not available for their local system or because there were problems with regard to synchronization of the medication data	“… the software provider does not offer the ARMIN software program yet …” (GP-23)
		Some interviewees feared that an additional software program could slow down their local computer system or make it more failure-prone	
Cost	Additional costs	Some CPs stated that it was expensive to participate because they would have to employ additional staff	“… the initial intervention of the medication review is very time-consuming and cannot be implemented in the daily work routine, i.e., additional staff is needed …” (CP-2)
		Many CPs and GPs stated the costs were (too) high because they have to meet the technical requirements, e.g., buying new hardware and software to connect their computer system with the secured online medication server	
II. Outer setting			
Patient needs & resources	Lack of benefit	Some GPs believed they already provide optimal patient care which cannot be further improved by ARMIN MM	“… some kind of medication management is already offered, i.e., brown-bag and medication reviews… physicians are contacted when determining potential DRPs …” (CP-8)
		CPs argued that they already have a good overview of many patients’ medication in their pharmacy and that they can easily check for DRPs or even perform medication reviews	
Cosmopolitanism	Poor GP-CP communication	CPs reported that it can be tough to communicate DRPs to GPs, i.e., because they have different views on DRPs' relevance and because GPs are rarely available. CPs would like to have a better exchange with GPs about how to deal with specific drug interactions	“… there should be some kind of exchange with GPs on how to deal with relevant interactions, e.g., within a quality circle …” (CP-7)
Peer pressure	n.a		
External policy & incentives	n.a		
III. Inner setting			
Structural characteristics	n.a		
Networks & communication	n.a		
Culture	n.a		
Implementation climate	GP’s professional sovereignty	Many GPs saw their professional sovereignty in danger, many CPs described this potential threat to GPs as a major problem for their collaboration. One CP experienced this phenomenon especially with older GPs	“… GPs, especially older GPs, fear that their professional sovereignty would be curtailed …” (CP-4)
	Lack of participating GPs/CPs	GPs and CPs have to collaborate closely to perform joint MM. However, most CPs and GPs were not able to find a collaboration partner	“… there were almost no GPs in our vicinity who participated in the ARMIN project …” (CP-6)
		CPs reported that contacted GPs were not interested in participating in ARMIN	
	Lack of suitable patients	GPs reported that their patients were not fit for the service because they were too old or had cognitive disorders	“… the idea of ARMIN is a good, but the implementation is rather difficult, e.g., patients do not commit themselves to one GP and one pharmacy …” (GP-10)
		In ARMIN, patients have to commit to a fixed pharmacy-GP-pair. However, HCPs stated that many patients preferred to visit different pharmacies or GP practices instead	
	Other priorities	Several GPs and CPs stated they have prioritized other tasks	“… when taking over a new family practice, there was not enough time to implement this program …” (GP-1)
Readiness for implementation	Lack of time and training	Many GPs and CPs reported that they do not have enough time to provide an additional service or participate in programs such as ARMIN	“… the 1-day ARMIN seminar was too short, i.e., additional seminars were attended …” (CP-10)
		One CP felt the provided workshop was not sufficient for learning how to perform the MM	
	Poor internet connection	Some HCPs did not have access to fast internet (usually a connection via fiber optic cable network), particularly in rural areas	“… the internet is too slow for ARMIN and has to be upgraded …” (GP-27)
IV. Characteristics of individuals			
Knowledge & beliefs about the intervention	Interference by CPs	GPs had concerns about CPs interfering with the therapy of patients and the feeling that there would be some kind of surveillance through CPs, for example with regard to drug interactions.	“… pharmacists interfere all the time … feels like surveillance …” (GP-1)
	Success depends on existing GP-CP relationship	Whether ARMIN is able to improve patient care may depend on the relationship between GPs and CPs	“… patients are upset because CPs tell them there is something wrong about their prescription… it depends on how GPs and CPs work together …” (GP-4)
	Negative benefit-cost ratio	Some CPs and GPs considered the benefit–cost ratio of the project to be negative	“… previous pilot projects failed, e.g., they required too much time, there were too many meetings, and finally no outcomes …” (CP-8)
		One CP refused to participate in ARMIN because she had made negative experiences in another pilot project in which the benefit did not far outweigh the costs	
	Lack of data security	In ARMIN, the participants’ local computer systems are connected to the online medication server. Few GPs were concerned the medication server was not secure or feared that SHI companies are able to access their data	“… the ARMIN interfaced is called a SHI trojan… data from the local system may be transferred to the SHI fund… changing the interface might increase the willingness to participate …” (GP-14)
	Lack of knowledge and being misinformed	Some GPs had poor knowledge about ARMIN, e.g., what ARMIN is exactly about, how it would be implemented in daily routine and which potential benefit it might have. Further, some GPs even had wrong perceptions related to reimbursement, i.e., there was none, and technical aspects. Corresponding barriers were later neither checked by HCPs nor were additional information provided by SHI	“… there is no remuneration …” (GP-15)
	Time required for medication review	GPs estimated the time needed to conduct medication reviews shorter than CPs and much shorter than actually scheduled (i.e. approx. 90 min per medication review). Yet, even GPs who estimated 2–10 min for a medication review reported time as a barrier	Estimated time needed for a medication review [min] (mean ± SD; range): GPs: 21 (± 14; 2–60), CPs: 75 (± 48; 30–225)
Self-efficacy	n.a		
Individual stage of change	n.a		
Individual identification with organization	n.a		
Other personal attributes	Wait and see	Few GPs hesitated to participate because they were not sure how ARMIN will perform, whether it will ever be implemented in routine care and thus also be adequately remunerated	“… I need to be younger and more open to participate …” (GP-26)
		Some HCPs were just not open and motivated enough to participate	
	CPs’ fear of endangering GP-relationship	CPs considered interprofessional MM useful. However, some CPs were afraid they could threaten their relationship with GPs when they interfere the medication process	“… it is the GP’s area of responsibility… I do not force it to keep our good relationship …” (CP-13)
V. Process			
Planning	n.a		
Engaging	Lack of promoting and advertising the project	Some HCPs wished they had received more information about the project or to be contacted at all	“… we did not get enough information… no one approached us …” (CP-12)
	Lack of personal support	Some CPs and GPs would like to get more support from the corresponding SHI fund with regard to the initial set-up, e.g., questions about the contract, installation of hardware and software	“… someone should help to set everything up …” (GP-34)
	Lack of involving HCP in management positions	GPs who worked in a medical service centers mentioned that participation was impossible when their management did not approve or engage with the program	“… the SHI fund contacted me—but not the management who has to decide …” (GP-33)
Executing	n.a		
Reflecting & evaluating	n.a		

*ARMIN*: Arzneimittelinitiative Sachsen-Thüringen (medication management program); *CFIR*: consolidated framework for implementation research; *CP*: community pharmacist; DRP: drug-related problem; *GP*: general practitioner; *HCP*: healthcare professional; MM: medication management; SHI: statutory health insurance

## Discussion

### Statement of key findings

This study showed that recruiting and attracting HCPs to an interprofessional MMP is associated with a wide range of barriers at multiple contextual levels. Although GPs and CPs thought that ARMIN can improve patient care, several barriers stood in the way of their participation. Most barriers were related to collaboration between GPs and CPs, eligibility for participation, local circumstances, IT, and cost–benefit ratio.

### Interpretation

We observed that both GPs and CPs mentioned the same key barriers, which indicates that both HCPs struggle with similar problems. For example, both GPs and CPs could not find a collaborating CP or GP, respectively (CP-6). Although some HCPs approached potential partners, no collaboration in MMP could be initiated, which highlighted the need for external support especially during the phase of registration and finding a collaborating partner [33]. The lack of GP participation could have been at least partly the consequence of the lack of available and interoperable IT systems as reported by many GPs (GP-23). Furthermore, collaboration between GPs and CPs was a key barrier in this study, as many HCPs mentioned the GP’s professional sovereignty as a barrier (CP-4). Additionally, CPs reported communication and collaboration with GPs, especially when solving DRPs, to be challenging (CP-7). This finding aligns with the results of a systematic review on interprofessional collaboration between CPs and GPs which identified barriers such as negotiating professional boundaries and perceived skills and knowledge [34]. Hence, establishing a collaborative relationship between a GP and a CP before participating in an interprofessional MMP seems essential. GPs and CPs should initiate collaboration in less complex and easy-to-access services to “break the ice” and negotiate professional boundaries to improve GP-CP collaboration [34–36]. Also, CPs reported that older GPs were less likely to collaborate, which agreed with the findings of previous research [34].

Furthermore, GPs and CPs reported expenditure of time as a common barrier [37], even though the time usually required to perform a medication review was sometimes underestimated. As only GPs frequently mentioned other priorities as a barrier, we hypothesize that GPs prioritized other tasks over the MMP, possibly because GPs already have an established professional role in managing patients’ therapy in contrast to CPs who are trying to expand their professional role [38]. HCPs can also prefer other projects which are already implemented at the national level, or are about to be implemented in the near future (CP-6), or which are better remunerated. Hence, new programs must provide an added value in patient care or a financial advantage for HCPs. Furthermore, at the beginning of pilot projects GPs and CPs should be recruited as pairs to generate seed GP-CP pairs. Additional GPs and CPs can then join individually over the course of a project. Of course, seed GP-CP pairs cannot be generated everywhere, but, wherever successful, those pairs should be used as best-practice examples to promote the MMP. Moreover, costs and issues of software set-up as well as concerns about data security were often reported by GPs (GP-14), indicating major IT-related barriers and a low level of IT-related readiness for implementation. However, often barriers which emerged initially were neither verified later by the HCPs nor were HCPs contacted by the responsible SHI fund with periodic updates. Hence, wrongfully made assumptions and no longer existent problems could have negatively influenced the HCPs’ decision on participation. To overcome this barrier more effectively, proactive support for HCPs is needed. The SHI fund could follow up more frequently with HCPs and organize reference-visits at already participating family practices and pharmacies to demonstrate the general workflow and user-friendliness and alleviate concerns about IT-related practicability. Further, establishing certified and trusted standards for the provision of software solutions or entire e-health systems might reduce GPs’ concerns about data security and associated liability issues [39].

At the patient level, a low demand for MMPs was also revealed in this study as only one HCP (out of 51) was asked by a patient about ARMIN [40–42]. This shows that, besides HCPs, patients also need to be engaged and motivated to participate in a new program. Patients should therefore be sufficiently informed about a program and how they can benefit from it [43], thereby enhancing patient demand. Obviously, patients’ HCPs can inform and engage them. However, as recruiting patients was burdensome for HCPs, SHI funds or professional organizations should provide support, thus relieving individual HCPs of these resource-intensive tasks.

### Strengths and weaknesses

The following limitations merit mention. First, interviews were not audio-recorded but protocolled by two researchers, one of whom was focused on taking notes while the other one was conducting the interview. This technique seemed adequate as the results of each interview were compared and discussed by the researchers immediately afterwards and the interview guideline included a large fraction of closed questions. Second, participants’ potentially narrow knowledge about the MMP service might have limited their capability to answer specific questions sufficiently. To minimize the risk of asking too detailed or complex questions, the interview guide was comprehensively piloted. Further, the interviewee’s knowledge was explored at the beginning of each interview and, if needed, additional information on the ARMIN project was provided. Thirdly, recruitment was not exclusively performed by the researchers who were supported by the SHI fund when approaching potential participants. Although unusual, we deemed this approach to be the most promising one with regard to HCPs’ willingness to participate because HCPs have already known SHI funds’ employees but not the researchers. Even though it cannot be completely ruled out that HCPs who declined to participate when contacted by the researchers might have had differing attitudes, this seems unlikely because those HCPs mostly mentioned a shortage of time rather than their opposition to the ARMIN project in its entirety as the governing reason for not participating. Fourth, the data generation and analysis were solely performed by researchers with a pharmacy background. Yet a researcher with a medical background supported the conception of the study and reviewed the results and the conclusions drawn. In contrast to anonymously surveying HCPs, interviewing them might have generated social desirability bias. Still, HCPs were asked to elaborate their response to obtain responses as valid as possible. Lastly, our sample size comprised only 51 HCPs. On the one hand, this sample size seems sufficient to reach data saturation, but on the other hand it does not allow broad generalizability because HCPs were selected on the basis of their prior experience with other components of the ARMIN project. However, the sample was diverse with regard to age, sex, professional experience and local setting, i.e. region and workplace. Characteristics of these participants might allow for partial transferability to HCPs in similar settings, i.e. other states in Germany.

### Future research

On the one hand, this study identified several barriers to participation in an interprofessional medication management. On the other hand, it revealed that most interviewees advocate the ARMIN MMP. Future studies should close this gap by employing tailored implementation strategies and evaluating which strategies are most efficient. Potential strategies to be evaluated for fostering participation in future programs could include, first and foremost, the creation of new opportunities for collaboration and joint participation between GPs and CPs, then advertising a program to patients while highlighting the potential benefits, and, last but not least, addressing HCPs’ reservations regarding potential and sometimes wrongly perceived barriers over the course of the project.

## Conclusion

The generally positive attitudes of GPs and CPs towards the interprofessional MMP could be a great potential. However, key barriers such as GP-CP collaboration, the IT readiness of HCPs, and informing and motivating HCPs and patients, all need to be addressed in order to raise awareness of the service and increase participation.

## Supplementary Information

Below is the link to the electronic supplementary material.

Supplementary file1 (PDF 74 kb)

Supplementary file2 (PDF 199 kb)
