# Supplementary material for: Attitudes of non-participating general practitioners and community pharmacists towards interprofessional medication management in primary care: an interview study
Source: Int J Clin Pharm. 2022 Oct 8;44(6):1380–93. doi: 10.1007/s11096-022-01434-3 (PMC9547634; doi:10.1007/s11096-022-01434-3)
Supplement: Supplementary file 1 — Supplementary file1 (PDF 74 kb) [file 11096_2022_1434_MOESM1_ESM.pdf]

Robert Moecker[1,2], Andreas Fuchs[3], ARMIN Study Group[4], Walter E. Haefeli[1,2], Marina Weissenborn[1,2†], Hanna M. Seidling[1,2†*]

Interview guide for semi-structured interviews with general practitioners and community pharmacists

translated and abridged

Short introduction

- Why these interviews are conducted and how results will be used
- How the interview is structured
- Informing about voluntary participation and anonymous data analysis

Knowledge and opinion about ARMIN (Arzneimittelinitiative Sachsen-Thüringen; Depending on the level of knowledge, additional information was provided by the researchers.)

- What do you know about ARMIN? (e.g., goals, procedure, content of modules)
- What are positive and negative aspects about ARMIN?

Agreement with statements about the following topics and exploration why participants have this opinion or how they came to their answer.

- Topics/statements (table 2):
- Anything you would like to add?
- What would you need to participate in ARMIN?
