# Supplementary material for: Attitudes of non-participating general practitioners and community pharmacists towards interprofessional medication management in primary care: an interview study
Source: Int J Clin Pharm. 2022 Oct 8;44(6):1380–93. doi: 10.1007/s11096-022-01434-3 (PMC9547634; doi:10.1007/s11096-022-01434-3)
Supplement: Supplementary file 2 — Supplementary file2 (PDF 199 kb) [file 11096_2022_1434_MOESM2_ESM.pdf]

Robert Moecker[1,2], Andreas Fuchs[3], ARMIN Study Group[4], Walter E. Haefeli[1,2], Marina Weissenborn[1,2†], Hanna M. Seidling[1,2†*]

**Theme**	**Code**	**Example** (red = pharmacist, blue = general practitioner)
**Collaboration of GPs and CPs**	Professional sovereignty	CP would be too much involved in in therapie "CP intrudes therapy"
		Supervision by CPs
		GPs insist on professional sovereignty
	Communication	Difficulties in communication with CPs
		Communication with GPs is difficult and time-consuming
**Information technology**	Software	Technical "inconsistencies in the software"
		Software provider does not offer ARMIN software
		Technical "inconsistencies in the software”
	Failure proneness of the technical infrastructure	Additional software program (potentially prone to failure) is required + scanner device (MedPlan) is also another device
	Data security	KV-SafeNet is not secure
		Uncontrolled data flows, S3C interface is a "SHI fund trojan".
		Connection of the PVS (local software system) with AOK (SHI fund) "Can AOK read data from my PVS?"
		KV (Association of Statutory Health Insurance Physicians) should not have access to GP’s local software system
	Traceability of changes in the medication list	Questionable/limited traceability of who made what changes in the medication list (beyond the general distinction of GP and CP).
	User friendliness	Annoying pop-up windows triggered by medication catalogue recommending the cheapest drug
		Too little automation in the process, also in the issuing of medication lists
	Performance prescription scan	When looking for a drug, WiVO prescription scan is worse than typing in central pharmaceutical number (PZN)
**Eligibility for participation in ARMIN**	Modul 1 – WiVO (preferred generic prescribing)	Limited freedom of therapy/too much regulation (WiVO)
		WIVO exchange can lead to exchange of dosage forms which might not be adequate
	Modul 2 – MedKat (preferred prescribing of first-line drugs)	Medication catalog is not very individualized for single patients

	Distinct GP-pharmacy pairs	Allocation by GP-pharmacy pairs. With some GPs there may not be good communication
		Commitment to *one* pharmacy. Patients do not necessarily buy all OTC drugs in *one* pharmacy. OTC drugs are not completely covered
		Cooperation with pharmacy creates impression of advertising for pharmacy
	Requirements for participation (pharmacy)	CPs must be a member of State Association of Pharmacists.
		CPs must be a member of State Association of Pharmacists
	Requirements for participation (patients)	Patients need to take five or more drugs. Patient should be eligible if they are taking at least three drugs.
		Patients need insurance with AOK PLUS (patients from other SHI funds cannot participate)
		ARMIN is offered by *one* SHI fund only. This is too little harmonization.
	Continuation of a pilot project uncertain	Future duration and remuneration of a program are uncertain
	Complexity	Too complex to implement in practice
	Medication list (Bundesmedikationsplan)	Manufacturer column on medication list is confusing for patients
		Medication list should be stored on patients' insurance card
**Cost-benefit ratio**	Expenditure of time	High expenditure of time in general
		High expenditure of time for informing and recruiting patients
		Too much time required for exchange with pharmacy in medication management
		High expenditure of time in general
		High bureaucratic effort, "a lot of paperwork"
		High bureaucratic effort
	Cost	High costs in general
		Additional staff required
		Software is expensive
		Software is expensive
	Negative effort/benefit ratio	Effort and benefit are in negative proportion
	Effort pilot project	Particularly high effort as participant in a *pilot* project
	Little additional value	No outcome/benefit

		No additional benefit to existing patient care (CP already offers medication management)
		No additional benefit
		Little benefit
		The problem is patients do not adhere to the medication list. ARMIN has no potential to improve that.
**Local circumstances**	GP participation	Lack of participating GPs in the surrounding area
		GPs oppose ARMIN
		GP uses a scanner QR codes of medication lists. But no one else has one or uses it.
		Only few GPs participate
	CP participation	No competent participating pharmacies
	Up-to-dateness of software	GPs do not have up-to-date software
	Patient participation	Problem in general: Patients frequently change GP or pharmacy
	No suitable patients	Many older patients (75-90) with insufficient cognition
	Competing projects	In the future, possibly standardized services are remunerated.
		Another, competing project for already existing project (for GPs)
		Alternative project preferred
	Other priorities	There are problems with higher priority such lacking availability of particular medicines
	Internet infrastructure	poor internet infrastructure
		poor internet infrastructure in the countryside
	Experience with other pilot projects	Bad experiences in the past, e.g., electronic insurance card (eGK)
	Impairment daily work routine	Daily pharmacy routine is disrupted by high expenditure of time
**Minor theme: Misinformed/ wrong assumptions**	Remuneration	ARMIN is not remunerated
	IT	No interface available yet (it does not exist yet)
